# Genetics of Parenting: The Power of the Dark Side

**DOI:** 10.1037/a0035388

**Published:** 2013-12-23

**Authors:** Bonamy R. Oliver, Maciej Trzaskowski, Robert Plomin

**Affiliations:** 1 School of Psychology, University of Sussex, Brighton, United Kingdom, and Medical Research Council Social, Genetic, & Developmental Psychiatry Centre, King’s College London Institute of Psychiatry, London, United Kingdom; 2 Medical Research Council Social, Genetic, & Developmental Psychiatry Centre, King’s College London Institute of Psychiatry, London, United Kingdom

**Keywords:** behavior genetics, parental control, parental feelings, parental negativity, parental positivity

## Abstract

Reviews of behavioral genetic studies note that “control” aspects of parenting yield low estimates of heritability, while “affective” aspects (parental feelings) yield moderate estimates. Research to date has not specifically considered whether positive and negative aspects of parenting—for both feelings and control—may explain these etiological distinctions. We addressed this issue using parent reports of parenting in a large twin sample in the United Kingdom, at ages 9 (*N* = 2,260 twin pairs), 12 (*N* = 3,850 twin pairs) and 14 (*N* = 2,293 twin pairs) years. Our findings supported previous work indicating that parental feelings show greater heritability (*h*^2^) than control (across all ages, mean *h*^2^ feelings = .42, control = .13). Of specific interest is our novel finding that for control as well as for feelings, the heritability for negative aspects of parenting was greater than for positive aspects (e.g., across all ages, mean *h*^2^ total negativity = .44; total positivity = .12). Results across the 3 ages using common pathway models for all scales further endorsed our hypotheses. Previous research has shown that children’s genetically driven characteristics elicit parenting; our pattern of our results suggests that what is critical is the “dark” side of these characteristics for eliciting negativity from parents, whether feelings toward the child or control strategies are considered. Improving understanding of how the environment is shaped by the dark side is important theoretically and, ultimately, for targeting intervention.

As first described by Bell (1968), parenting is an interactional process in which child characteristics influence parenting behaviors as well as the other way around. In a then-controversial presidential address to the Society for Research in Child Development in 1991 (Scarr, 1992), behavioral genetic evidence was used to make the case that children’s experiences are dependent on their genetic propensities. Three types of so-called genotype-environment correlation have been described (Plomin et al., 1977). Passive gene–environment correlation refers to associations between child genotype and environmental exposure; that is, parents pass on genetic material as well as creating a home environment for their children based on their own (heritable) characteristics. Evocative genotype–environment correlation involves the child’s elicitation of environmental experience as a function of his or her genotype. Finally, active genotype–environment correlation suggests that children are active agents in their own socialization, including parenting, and this active manipulation of environmental experience is, at least in part, genetically determined. Despite a plethora of previous parenting research using behavioral genetic approaches to disentangle these effects (see Knafo & Jaffee, 2013), the field has thus far largely confounded parenting dimensions and their valence (positive or negative), potentially masking important underlying architectural distinctions. For the first time, we systematically examined positive and negative aspects of two parenting dimensions, parental feelings and parental control, in late childhood to early adolescence.

## Genetics of Parenting

Behavioral genetic studies of parenting are informative since they offer unique insights into the contributions of parent and child characteristics; the twin design can be useful in this context, comparing identical, or monozygotic (MZ), and fraternal, or dizygotic (DZ), twins. So-called *parent-based twin designs* compare the parenting behaviors of twin parents, with the premise that to the extent that parenting behaviors shown by MZ twin parents toward their offspring are more similar than those shown by DZ twin parents, genetically influenced parental characteristics are implicated. A child-based study, like the current one, has twin children as the focus for analyses. Here, the extent to which MZ twins are similarly parented compared with DZ twins is evidence of significant genetic influence on aspects of parenting but suggests that parental behavior reflects genetically influenced characteristics of the child, such as their temperament.

## Parental Feelings and Control

Subsequent theoretical and empirical perspectives on parenting have remained largely founded in Baumrind’s earlier work on parenting styles, which at its core, focused attention on two key aspects of parenting—responsiveness/warmth and demandingness/control (Baumrind, 1973). While researchers have distinguished aspects of parenting further, most notably in the area of parental control (e.g., Barber & Harmon, 2002) and have varied in their construct labels, these two broad dimensions have been endorsed through numerous studies that have sought to characterize them (e.g., Barber, Stolz, & Olsen, 2005; Cummings, Davies, & Campbell, 2000; Dodge, Pettit, & Bates, 1994; Maccoby & Martin, 1983; Rothbaum & Weisz, 1994). Here, we have conceptualized these parenting dimensions as parental feelings (warmth, closeness, hostility, frustration) and parental control (discipline strategies such as remaining firm and the use of physical discipline); these dimensions have shown robust modest to moderate associations to children’s outcomes (e.g., Parke & Buriel, 2006).

Reviews of behavioral genetic studies have noted that control aspects of parenting tend to yield low estimates of heritability while parental feelings yield moderate estimates (Kendler & Baker, 2007; Plomin, 1994; Rowe, 1981, 1983). To be clear, in child-based studies, these findings suggest that genetically influenced child characteristics may be more important for eliciting parental feelings than control. However, research has seldom distinguished between positive and negative parental feelings and particularly between positive and negative control strategies. Blurring the positive and negative sides of feelings and control may mask important underlying foundations of parenting. Harsh discipline and effective supervision, for example, may not be opposite ends of a single continuum, and neither may hostility and warmth (see Pettit & Arsiwalla, 2008). Thus, we hypothesized that the underlying genetic architecture of these aspects of parenting may also be distinct. Specifically, following existing relevant family research (e.g., Kendler & Baker, 2007; Rasbash, Jenkins, O’Connor, Tackett, & Reiss, 2011) as well as work outside the field (e.g., Baker, Cesa, Gatz, & Mellins, 1992; Baumeister, Bratslavsky, Finkenauer, & Vohs, 2001), we predicted that negativity would show greater heritability than positivity across parental feelings and control as well as within parental feelings and within control.

For the first time in a large-scale, longitudinal, child-based twin study, we explicitly compared the genetic and environmental etiology of positivity and negativity, positive and negative feelings, and positive and negative control.

## Method

### Sample and Procedure

The sampling frame for the current study was the Twins Early Development Study (TEDS), a population-based, longitudinal study of twins born in England and Wales in 1994–1996, recruited from U.K. birth records. TEDS has been demonstrated to be reasonably representative of the U.K. population (e.g., Harlaar, Spinath, Dale, & Plomin, 2005), acknowledging that literacy is a requirement for completing the questionnaires. For example, for families with 12-year data, 93.5% of the mothers in the sample self-reported ethnicity as White and 43.6% had qualifications at A-level or higher (the national educational qualification taken at age 18 years in the United Kingdom); the equivalent U.K. population percentages for this generation are 93% White and 32% for qualifications at A-levels or higher (Walker, Maher, Coulthard, Goddard, & Thomas, 2001). TEDS is described in detail elsewhere (see Haworth, Davis, & Plomin, 2013; Oliver & Plomin, 2007; Trouton, Spinath, & Plomin, 2002). Data at all ages were collected through parent questionnaires sent to families by mail. For the current study, at each age, we selected same-sex twin pairs only and excluded all twin pairs where either twin had parent-reported medical or neurological conditions. Note that due to differences in study procedures, data available differed at each age. Since this brief report does not include longitudinal analyses, we took the decision to include all appropriate data available at each age such that while some families were included at all ages, some families were included at only one or two ages. Thus, the current study included 2,260 twin pairs at age 9 (1,202 MZ and 1,058 DZ; 1,034 boys and 1,226 girls), 3,850 twin pairs at age 12 (2,027 MZ and 1,823 DZ; 1,752 boys and 2,098 girls), and 2,293 twin pairs at age 14 (1,231 MZ and 1,062 DZ; 1,028 boys and 1,265 girls). We determined zygosity using parent ratings of physical similarity shown to be more than 95% accurate compared with DNA testing (see Price et al., 2000). For cases where zygosity was unclear, DNA testing was conducted to confirm zygosity. TEDS research was approved by the Institute of Psychiatry Ethics Committee, and all participants gave informed consent.

### Measures

We generated eight scales from identical parent-report measures at child ages 9, 12, and 14 years of parental feelings and control. For feelings, we used an adapted short form (seven items) of the Parental Feelings Questionnaire (PFQ; Deater-Deckard, 2000) and for control, a short (four-item) discipline (parenting strategies) questionnaire adapted from Deater-Deckard, Dodge, Bates, and Pettit (1998). Item responses were *rarely/never* (0), *sometimes* (1), and *often* (2), and coding was reversed for items as necessary. Two standard composite measures were created at each age from the PFQ and Discipline questionnaires: *Feelings* from seven PFQ items, including the three positive (e.g., “I feel close to my child”) and four negative items (e.g., “I feel frustrated by my child”) and *Control* comprising four discipline items including two positive (e.g., “I am firm and calm with him or her”) and two negative (e.g., “I tell him or her off or shout at him or her”) items. In addition, scales were generated at each age to depict *Negative Feelings* (four negative PFQ items), *Positive Feelings* (three positive PFQ items), *Negative Control* (two negative discipline items), and *Positive Control* (two positive discipline items). We then created composite measures for all negative items (*Negativity:* four negative PFQ items, two negative discipline items) and for all positive items (*Positivity*: three positive PFQ items, two positive discipline items). For each scale, items were summed and averaged by the number of items with positive/negative valence and control/feelings elements as appropriate.

Although face validity for our scales is reasonable and appropriate for the hypothesis-driven nature of the current report, variable internal consistency for these scales was found, with reliabilities lower for scales with fewer items, as is to be expected: 9 year: Feelings α = .68 (Negative feelings α = .75, Positive feelings α = .45), Control α = .44 (Negative control α = .33, Positive control α = .61), Negativity α = .74, and Positivity α = .51; 12 year: Feelings α = .70 (Negative feelings α = .75, Positive feelings α = .50), Control α = .41 (Negative control α = .29, Positive control α = .63), Negativity α = .74, and Positivity α = .52; 14 year: Feelings α = .72 (Negative feelings α = .75, Positive feelings α = .57), Control α = .36 (Negative control α = .25, Positive control α = .63), Negativity α = .75, and Positivity α = .56).

### Analyses

#### Data preparation

The effects of children’s age and sex on measures were minimal, accounting for no more than 1% of the variance for any scale at any age. Nevertheless, standardized residual scores (controlling for age and sex) were used in the main analyses as is standard practice in twin studies to ensure that twin correlations are not artificially inflated due to the children being the same age and sex (McGue & Bouchard, 1984). All of the measures were somewhat skewed, with positive feelings the most skewed at each age (9 years skew = 1.93; 12 years skew = 2.03; 14 years skew = 1.85; further details of skew on all measures are available on request). To avoid violation of the normality assumption implicit in the analyses involved, we applied Van der Waerden rank transformation to our measures (Lehmann, 1975). All analyses were conducted on these transformed data.

#### Twin analyses

The classic twin method allows the decomposition of phenotypic variance into additive genetic (A), shared (C), and nonshared (E) environmental components (e.g., Plomin, DeFries, Knopik, & Neiderhiser, 2013). Twin intraclass correlations (ICCs) provided an initial approximation of these ACE contributions. Heritability can be estimated as twice the difference between MZ and DZ ICCs, and shared environment as the difference between the MZ correlation and the heritability estimate. Estimates of the nonshared environmental component include measurement error; it is the only source of variance responsible for MZ twin differences and thus is estimated as the extent to which the MZ ICC is less than 1. Structural equation models provide more elegant estimates of these variance components as well as confidence intervals and model fit indices.

**Figure 1 fig1:**
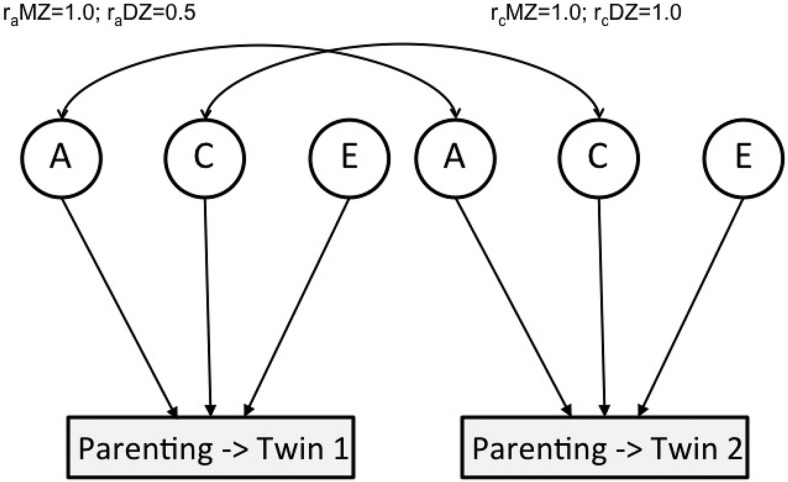
Univariate individual differences model fitting: A = additive genetic influence; C = shared environment; E = nonshared environment; paths a, c, and e = effects of A, C, and E on the quantitative trait. Genetic relatedness, or the genetic correlation (r_a_), is 1.0 for identical, or monozygotic (MZ), twins and 0.5 for fraternal, or dizygotic (DZ), twins; environmental correlation (r_c_) is assumed to be 1.0 both for MZ and DZ twins.

As well as fitting the standard univariate twin model (see Figure 1) for each of our scales across all ages, because the reliability of some scales was not optimal we further tested our hypotheses using the common pathway model.[Fig-anchor fig1]

**Figure 2 fig2:**
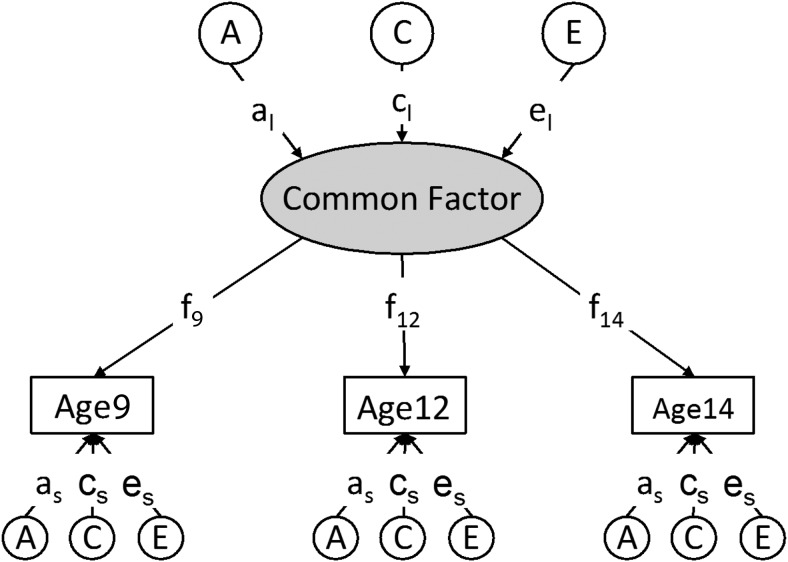
Common pathway model: a_l_, c_l_, and e_l_ represent additive genetic (A), shared environment (C) and nonshared environmental (E) components of the latent factor variance across the three ages; paths f_9_, f_12_, and f_14_ are the factor loadings from measures at each age; a_s_, c_s_, and e_s_ are the specific additive genetic, shared environmental, and nonshared environmental influences on scales at each age.

In this model (see Figure 2), all common genetic and environmental variance is mediated through a single latent factor with age-specific factor loadings that allow influences on change over time (Martin & Eaves, 1977). The common latent factor includes the common variance across the ages and is thus more reliable than the scales at each age. The common pathway model allows each phenotypic variable to be also influenced by specific environmental or genetic factors that are not shared across age, which we refer to as *age-specific effects*.[Fig-anchor fig2]

Model fitting and all subsequent analyses were done using OpenMx (Boker et al., 2011), which uses minus twice log likelihood (–2LL) as the evaluation of the fit. To verify how well an alternative model fits the data, a relative comparison of the fit statistic (–2LL) is made between the saturated model (the baseline model) and the model of interest (here, the common pathway model). Given that the difference between these statistics follows the chi-square distribution, we used the chi-square test with degrees of freedom equal to the difference between the number of parameters of each model. However, due to the sensitivity of this index to sample size, indices such as the Bayesian information criterion (BIC; Schwarz, 1978) or the Akaike information criterion (AIC; Akaike, 1987) are commonly used in behavioral genetic research (e.g., Kendler, Gardner, & Lichtenstein, 2008), with suggestions that BIC performs better with larger sample sizes and more complex models (Markon & Krueger, 2004). The lower the value of these indices, the better the balance of explanatory power and parsimony indicates better fit.

## Results

### Preliminary Analysis

For information, descriptive statistics for all raw measures are included in Table 1 by sex and zygosity for each age.[Table-anchor tbl1]

### Genetic Analysis

As a first step in estimating genetic and environmental influence, twin ICCs were calculated separately for the MZ and DZ twins (see Table 2). In all cases, MZ twin similarity exceeds DZ twin similarity, indicating some genetic influence. The DZ twin correlations are greater than half the MZ twin correlations, indicating substantial shared environmental influence. Finally, the high MZ correlations indicate a minor role for nonshared environmental factors.[Table-anchor tbl2]

Results from the model-fitting analyses (shown in Table 3) were highly similar to those gleaned from the ICCs: genetic influence was significant for all scales, shared environment was substantial, and nonshared environment was negligible especially after discounting error of measurement. The focus of the present analysis was on heritability comparisons between feelings and control on the one hand and negativity and positivity on the other. As a prelude, the standard scales for PFQ Feelings and Control yielded a result to be expected from the literature: At all three ages, parental feelings were significantly more heritable than parental control, as indicated by the nonoverlapping 95% confidence intervals for the Feelings and Control scales. The average heritability across the three ages was 42% for parental feelings and 13% for parental control. As an aside, such comparisons were robust for negative feelings (44%) compared with negative control (27%) and for positive feelings (26%) compared with positive control (6%).[Table-anchor tbl3]

Nonetheless, returning to our study focus, we found across constructs that negative aspects of parenting are significantly more heritable than positive aspects, again at all three ages. For example, for negative and positive feelings, negative feelings showed significantly more heritability than positive feelings, with average heritabilities across the three ages of 44% and 26%, respectively; the pattern was similar for parental control, with average heritabilities across the three ages of 27% and 6% for negative and positive aspects, respectively. Finally, creating scales for all the negativity items and all the positivity items regardless of whether they were on the Feeling or Control scale yielded significantly greater heritability for the negativity than for the positivity, with average heritabilities across the three ages of 44% and 12%, respectively.

Acknowledging the fact that the reliabilities of some scales were less than desirable, we examined our hypothesis further by comparing saturated and common pathway models for each of our eight scales, Feelings, Control, Negative Feelings, Positive Feelings, Negative Control, Positive Control, Negativity, and Positivity across the three ages, thus exploiting the stability of a latent factor for these analyses. The results for this common pathway model (see Figure 2) are shown in Table 4, which includes model fit statistics and parameter estimates. In every case, the common pathway model was found to fit the data at least equally as well as the saturated model, providing a statistical rationale for using the model.[Table-anchor tbl4]

The pattern of heritability differences gleaned from the latent factors in these models remained the same as those for our univariate analyses of each scale at each age, adding further support for our hypothesis. That is, while latent factors for feelings and control differed significantly in their heritability in line with previous research (.38 and .08, respectively), there was additional emphasis on the distinctions between negativity and positivity. Specifically, for both feelings and control, negativity consistently yielded significantly higher heritability estimates than did positivity, a finding that held for the overall negativity and positivity latent factors (*h*^2^ = .42 and .10, respectively). The pattern of heritability estimates for each of the individual scales calculated from the common pathway model did not differ from those reported in our main univariate analyses. Factor loadings suggested that although age 12 had the strongest influence on the latent factors, all three ages were of similar magnitude. Note that since they were not central to the focus of study (in the interests of space in this brief report), the coefficients of the specific pathway are not reported here but are available from the first author.

## Discussion

In the current study, the etiology of positive compared with negative aspects of parental feelings and control was explicitly examined for the first time. A clear pattern of results emerged: the negative side of parenting showed significantly greater genetic influence than the positive side, regardless of whether parental feelings or control were assessed. It is important to note that these results replicated across ages 9, 12, and 14 years, as well as in common pathway models across the 3 years in acknowledgment of the variable reliability of our measures and to add confidence in our findings, with all contrasts between positive and negative aspects of parenting significant in our sample.

Previous reviews have highlighted the lower heritability of parental control compared with parental feelings, suggesting that genetically influenced child traits may be more reflected in parental feelings than in parenting control strategies (Kendler & Baker, 2007; Plomin, 1994; Plomin & Bergeman, 1991). Further, it has been suggested that parental control encompasses more learned, socially influenced parenting behaviors, whereas parental feelings are more strongly influenced by child characteristics such as temperament and behavior problems (e.g., Kendler & Baker, 2007). We too find that feelings show greater heritability than control over all. However, in line with our predictions, we have shown negative control to have greater genetic influence than positive control and negative feelings to have greater genetic influence than positive feelings. We argue that the “dark” side of genetically driven child characteristics plays a bigger role in eliciting parental negativity than do other child characteristics in eliciting positivity across feelings and control. For example, parental negativity encompassing hostility and harsh parenting may be more responsive to genetically driven challenging child temperament than positive features such as warmth and calmness are to less challenging traits. Although positivity versus negativity was not an explicit research question in previous studies, these features lend support to our findings (Kendler & Baker, 2007). Further, a recent study using genetically informed social relations models indicates consistency in negative responses elicited by individuals in the family and the importance of genetics for individual effects for negativity (Rasbash et al., 2011). Distinctions of parenting valence seem to be important for understanding family processes.

One of the earliest studies with relevant data was a study of 850 pairs of high-school twins (Loehlin & Nichols, 1976). In this study, parental control comprised items such as “stricter discipline” that showed little genetic influence, yet there was one important exception—an item involving spanking, which showed considerable genetic influence. Indeed, other studies have similarly shown harsh parenting to reflect genetic influence (e.g., Jaffe et al., 2004). This anomaly in heritability estimates could be due to harsh parenting involving more negative affect than other kinds of control (see Plomin & Bergeman, 1991). Notably, there is evidence outside the field of parenting of higher heritability for negative compared with positive affect (e.g., Baker et al., 1992). The reason that other studies have shown control scales to show less genetic influence than feelings scales may be that the control scales concerned have fewer negative-affective items. In the current study, our Control scale included negative-affective aspects of control.

One caveat is critical here. In categorizing measures of parenting into positive versus negative valence, we do not include maltreatment. That is, the pattern we report includes aspects of harsh discipline, such as yelling and spanking, but not abusive forms of parenting. In one study that explicitly looked at this distinction, Jaffee et al. (2004) found that while harsh discipline was moderately genetically influenced (25%), physical maltreatment was not (7%). These findings suggest that children’s genetic influences are largely irrelevant for their vulnerability to maltreatment and that characteristics of the perpetrator are what are important.

Although not a focus for this brief report, it is pertinent to highlight an additional finding of interest in the current study—that of a considerable shared environmental component both for control overall compared with feelings and for positive compared with negative aspects of parenting. In child-based designs like this one, shared environmental components indicate the extent to which parenting is consistent across children within the family. Our findings suggest that parents report being consistent in their discipline—particularly positive strategies—across members of the twin pair, as well in their feelings of warmth toward their children, once genetic similarities are accounted for.

We acknowledge limitations of the current study, such as the fact that the same parent rated both twins, likely to contribute to the high shared environment estimates in particular. The shared environmental estimates we find here mirror the genetic results in that higher shared environmental influence was found for control than feelings and for positivity than negativity. Although this may reflect genuine similarity in parental treatment strategies and warmth, it is possible that it is a parent reporting bias: parents may be more reluctant to say that they use different control strategies for their children or to admit to having more positive feelings about one twin than another.

We choose to use the simplicity of cross-sectional analyses to illustrate a novel path for parenting researchers within this brief report, with a view to offering a potential steer for more complex research. The robustness of our cross-sectional findings across age supports our perspective, but the findings are, by nature, limited. We acknowledge that the measures we used here are brief and not designed for the question in hand; indeed, the reliability of some our scales is variable, and this could have an impact on findings. Potentially, applying a more exploratory, factor analytical approach to our parenting variables would suggest they fall together differently. However, to evaluate our hypothesis-driven, conceptual question in this large sample, it was necessary to generate our scales a priori. Further, our common pathways approach capitalized on the increased reliability of the latent factor and yielded findings that additionally endorsed our hypotheses; moreover, the similarity of findings from our univariate and common pathways models suggests that the results from the former were robust to the lower reliabilities. However, we emphasize that more detailed measures across multiple methods and informants, as well as longitudinal study, will be critical for teasing out the issues we have raised. While stressing this need for replication, we posit that our results highlight an important new angle for understanding the mutuality of child and parent influences on the parent–child relationship. In turn, our findings suggest several key directions for research, such as extending current multivariate behavioral genetic research that has examined child characteristics important for parenting (Knafo & Jaffee, 2013) to identify specific associations between positive and negative child characteristics with parental negativity and positivity across control and feelings. Using parent-based designs would also be of interest to examine whether the dark side of genetically influenced parent characteristics plays similarly into the heritability of negativity and positivity in parent–child relationships. Further, another important question is the extent to which our findings tie in to contemporary intervention research that suggests that negative child characteristics may have a role in the effectiveness of interventions that aim to promote parental positivity (see Mash & Barkley, 2006; Sandler, Schoenfelder, Wolchik, & MacKinnon, 2011). Ultimately, studies that succeed in teasing out such child and parent effects could have key implications for informing parenting interventions. Finally, we suggest that our findings have considerable implications for work in other areas such as sibling and marital relationships, since negativity in these relations are likely to be similarly more genetically influenced than positivity and thus related to genetically influenced characteristics of the members of the dyad.

We hope that the findings of this study will be a springboard for discussion, offering potential new perspectives on classic questions in developmental psychology. We argue that child characteristics may be especially important for influencing negative aspects of parenting, for control as well as for feelings, but emphasize that further work with multiple measures, methods, and informants is needed. Better understanding how the environment is shaped by genetically driven individual differences in children’s characteristics is critical for basic science in developmental psychology and, ultimately, for targeting interventions.

## Figures and Tables

**Table 1 tbl1:** Descriptive Statistics for All Measures by Zygosity: Means (SDs)

Measure	Age 9 years					Age 12 years					Age 14 years				
	All (2,257–2,260)	Boys (1,032–1,034)	Girls (1,224–1,226)	MZ (1,200–1,203)	DZ (1,055–1,058)	All (3,831–3,850)	Boys (1,743–1,752)	Girls (2,094–2,099)	MZ (2,016–2,027)	DZ (1,820–1,825)	All (2,286–2,293)	Boys (1,026–1,028)	Girls (1,258–1,265)	MZ (1,229–1,231)	DZ (1,056–1s062)
Feelings	0.87 (0.55)	0.91 (0.56)	0.84 (0.55)	.088 (0.56)	0.86 (0.55)	0.82 (0.58)	0.87 (0.59)	0.79 (0.57)	0.81 (0.58)	0.84 (0.57)	0.82 (0.61)	0.85 (0.61)	0.80 (0.60)	0.82 (0.60)	0.82 (0.61)
Control	1.21 (0.61)	1.24 (0.63)	1.18 (0.59)	1.22 (.060)	1.19 (0.62)	1.08 (0.57)	1.12 (0.57)	1.05 (0.56)	1.10 (0.58)	1.06 (0.56)	1.02 (0.56)	1.04 (0.56)	1.00 (0.55)	1.04 (0.56)	0.99 (0.55)
Negative feelings	0.72 (0.44)	0.75 (0.45)	0.70 (0.43)	0.73 (0.44)	0.71 (0.43)	0.65 (0.44)	0.69 (0.44)	0.62 (0.43)	0.65 (0.44)	0.66 (0.43)	0.61 (0.44)	0.64 (0.44)	0.60 (0.45)	0.61 (0.44)	0.62 (0.45)
Positive feelings	1.85 (0.26)	1.84 (0.27)	1.86 (0.25)	1.85 (0.26)	1.85 (0.26)	1.83 (0.09)	1.82 (0.30)	1.84 (0.28)	1.84 (0.80)	1.82 (0.29)	1.79 (0.32)	1.79 (0.33)	1.80 (0.31)	1.80 (0.32)	1.79 (0.32)
Negative control	0.78 (0.38)	0.81 (0.39)	0.76 (0.37)	0.80 (.038)	0.77 (0.38)	0.66 (0.33)	0.69 (0.34)	0.63 (0.32)	0.67 (0.34)	0.64 (0.33)	0.63 (0.33)	0.67 (0.34)	0.60 (0.32)	0.65 (0.34)	0.61 (0.32)
Positive control	1.57 (0.44)	1.56 (0.44)	1.58 (0.43)	1.57 (0.43)	1.57 (0.44)	1.57 (0.44)	1.57 (0.44)	1.58 (0.44)	1.57 (0.45)	1.58 (0.43)	1.61 (0.44)	1.63 (0.44)	1.60 (0.44)	1.61 (0.43)	1.62 (0.44)
Negativity	1.50 (0.68)	1.55 (0.70)	1.46 (0.66)	1.53 (0.69)	1.47 (0.66)	1.31 (0.65)	1.38 (0.66)	1.25 (0.63)	1.32 (0.65)	1.30 (0.64)	1.25 (0.67)	1.31 (0.68)	1.20 (0.65)	1.26 (0.67)	1.23 (0.66)
Positivity	3.42 (0.55)	3.40 (0.57)	3.44 (0.53)	3.43 (0.54)	3.42 (0.56)	3.40 (0.56)	3.39 (0.56)	3.41 (0.56)	3.41 (0.57)	3.40 (0.56)	3.41 (0.59)	3.41 (0.59)	3.40 (0.58)	3.40 (0.58)	3.41 (0.59)
*Note*. Sample sizes given (in parentheses below the column headings) are twin pairs; for all measures, scores represent the average item score such that a mean of 0 indicates an average item score representing *rarely/never* and a mean of 2 indicates an average item score representing *often*. MZ = monozygotic; DZ = dizygotic.															

**Table 2 tbl2:** Monozygotic (MZ) and Dizygotic (DZ) Intraclass Correlations (ICCs) and Model-Fitting Estimates of Genetic (A), Shared (C) and Nonshared (E) Environmental Variance Components Across Scales and Ages

Measure	Age 9 years					Age 12 years					Age 14 years				
	ICC		A [CI]	C [CI]	E [CI)	ICC		A [CI]	C [CI]	E [CI]	ICC		A [CI]	C [CI]	E [CI]
	MZ	DZ				MZ	DZ				MZ	DZ			
Feelings	0.91	0.68	.47 [.41, .53]	.45 [.38, .50]	.09 [.08, .10]	0.90	0.72	.35 [.31, 39]	.55 [.51, .59]	.10 [.10, .11]	0.90	0.69	.43 [.37, .49]	.47 [.41, .53]	.10 [.09, .11]
Control	0.97	0.91	.13 [.11, .15]	.85 [.83, .86]	.03 [.02, .03]	0.96	0.91	.12 [.11, .14]	.84 [.83, .86]	.04 [.03, .04]	0.97	0.90	.14 [12-, 16]	.83 [.81, .85]	.03 [.03, .04]
Negative feelings	0.91	0.66	.50 [.44, .57]	.41 [.34, .47]	.09 [.08, .10]	0.89	0.69	.39 [.35, .44]	.49 [.45, .53]	.12 [.11, .12]	0.9	0.67	.44 [.39, .51]	.45 [.39, .51]	.10 [.09, .11]
Positive feelings	0.91	0.79	.25 [.21, .29]	.67 [.62, .70]	.09 [.08, .09]	0.91	0.79	.24 [.21, .27]	.67 [.64, .70]	.09 [.08, .10]	0.92	0.77	.30 [.26, .35]	.62 [.57, .66]	.08 [.07, .09]
Negative control	0.96	0.81	.31 [.28, .35]	.65 [.61, .68]	.04 [.04, .05]	0.94	0.81	.26 [.24, .29]	.68 [.65, .70]	.06 [.06, .07]	0.94	0.82	.24 [.21, .28]	.70 [.66, .73]	.06 [.05, .06]
Positive control	0.99	0.97	.04 [.04, .05]	.95 [.94, .95]	.01 [.01, .01]	0.98	0.94	.08 [.07, .09]	.90 [.89, .91]	.02 [.02, .02]	0.99	0.96	.06 [.05, .07]	.93 [.92, .94]	.01 [.01, .01]
Negativity	0.93	0.69	.47 [42, .53]	.46 [.40, .51]	.07 [.06, .07]	0.91	0.71	.41 [.37, .45]	.51 [.46, .55]	.09 [.08, .10]	0.92	0.7	.43 [.38, .49]	.49 [.43, .54]	.08 [.07, .09]
Positivity	0.96	0.92	.08 [.07, .10]	.88 [.86, .89]	.04 [.03, .04]	0.96	0.9	.10 [.09, .12]	.85 [.84, .87]	.05 [.04, .05]	0.97	0.89	.16 [.14, .19]	.80 [.78, .82]	.03 [.03, .04]
*Note*. CI = 95% confidence intervals.															

**Table 3 tbl3:** Model Fit Statistics for All Univariate Models Using OpenMx

Measure	Age 9 years				Age 12 years				Age 14 years			
	−2LL	*df*	AIC	BIC	−2LL	*df*	AIC	BIC	−2LL	*df*	AIC	BIC
Feelings	9979.73	4514	951.73	−29571.33	17026.28	7682	1662.28	−50282.35	10129.32	4568	993.32	−29894.87
Control	7379.56	4508	−1636.44	−32118.92	13087.14	7685	−2282.86	−54247.78	7879.48	4579	−1278.52	−32241.09
Negative feelings	10030.12	4514	1002.12	−29520.93	17346.69	7684	1978.69	−49979.47	10262.71	4574	1114.71	−29814.05
Positive feelings	9438.07	4517	404.07	−30139.26	16232.04	7694	844.04	−51181.74	9703.88	4571	561.88	−30346.59
Negative control	8705.75	4512	−318.25	−30827.78	15266.72	7689	−111.28	−52103.25	9125.64	4583	−40.36	−31029.98
Positive control	5529.89	4514	−3498.14	−34021.19	11253.52	7687	−4120.48	−56098.92	5538.73	4579	−3619.27	−34581.84
Negativity	9602.69	4509	584.69	−29904.55	16700.89	7668	1364.89	−50485.08	9923.17	4574	775.17	−30153.59
Positivity	7695.63	4513	−1330.37	−31846.66	13768.72	7676	−1583.28	53487.34	8070.73	4567	−1063.27	−31944.70
*Note*. −2LL = minus twice log likelihood; *df* = degrees of freedom; AIC = Akaike information criterion; BIC = Bayesian information criterion.												

**Table 4 tbl4:** Model Fit Statistics and Parameter Estimates for Common Pathway Models Using OpenMx

Latent factor	Saturated model				Common pathway				Coefficients		
	−2LL	*df*	AIC	BIC	−2LL	*df*	AIC	BIC	A [CI]	C [CI]	E [CI]
Feelings	34764.07	16722	1320.07	−111751.80	34817.73	16759	1299.73	−112022.34	.38 [.33, .44]	.58 [.52, .63]	.04 [.03, .05]
Control	27022.39	16730	−6437.61	−119563.58	27089.87	16767	−6444.13	−119820.29	.08 [06, .10]	.91 [.89, .93]	.01 [.00, .01]
Negative feelings	34973.88	16730	1513.89	−111612.09	35020.71	16767	1486.71	−111889.45	.44 [.39, .51]	.51 [.45, .57]	.04 [.04, .05]
Positive feelings	34240.45	16740	760.45	−112433.14	34272.38	16777	718.38	−112725.40	.29 [23, .35]	.67 [.62, .72]	.04 [.03, .05]
Negative control	31429.89	16742	−2054.11	−115261.23	31482.87	16779	−2075.13	−115532.44	.21 [.17, .24]	.78 [.75, .82]	.01 [.01, .01]
Positive control	21833.77	16738	−11642.23	−124822.30	21893.82	16775	−11656.18	−125086.44	.03 [.02, .05]	.96 [95, .98]	.01 [.00, .01]
Negativity	33415.55	16709	−2.45	−112986.43	33471.51	16746	−20.49	−113254.65	.42 [.37, .47]	.54 [.49, .59]	.04 [03, .04]
Positivity	28688.11	16714	−4739.89	−117757.67	28730.80	16751	−4771.20	−118039.17	.10 [.07, .13]	.89 [.86, .91]	.02 [.01, .02]
*Note*. −2LL = minus twice log likelihood; *df* = degrees of freedom; AIC = Akaike information criterion; BIC = Bayesian information criterion; CI = confidence interval.
